# Ocular Surface Disorders in Intensive Care Unit Patients

**DOI:** 10.1155/2013/182038

**Published:** 2013-10-29

**Authors:** Tuba Berra Saritas, Banu Bozkurt, Baris Simsek, Zeynep Cakmak, Mehmet Ozdemir, Alper Yosunkaya

**Affiliations:** ^1^Anesthesiology and Reanimation Department, Meram Medical Faculty, Necmettin Erbakan University, 42080 Konya, Turkey; ^2^Ophthalmology Department, Faculty of Medicine, Selcuk University, 42130 Konya, Turkey; ^3^Ophthalmology Department, Bitlis Government Hospital, 13000 Bitlis, Turkey; ^4^Microbiology Department, Meram Medical Faculty, Necmettin Erbakan University, 42080 Konya, Turkey

## Abstract

Patients in intensive care units (ICU) are at increased risk of corneal abrasions and infectious keratitis due to poor eyelid closure, decreased blink reflex, and increased exposure to pathogenic microorganisms. The aim of this retrospective study was to evaluate the ocular surface problems in patients who stayed in ICU more than 7 days and were consulted by an ophthalmologist. There were 26 men and 14 women with a mean age of 40.1 ± 18.15 years (range 17–74 years). Conjunctiva hyperemia, mucopurulent or purulent secretion, corneal staining, and corneal filaments were observed in 56.25%, 36.25%, 15%, and 5% of the eyes, respectively. Keratitis was observed in 4 patients (10%) who were treated successfully with topical antibiotics. Mean Schirmers test results were 7.6 ± 5.7 mm/5 min (median 6.5 mm/5 min) in the right, and 7.9 ± 6.3 mm/5 min (median 7 mm/5 min) in the left eyes. Schirmers test results were <5 mm/5 min in 40% of the subjects. The parameters did not show statistically significant difference according to mechanical ventilation, sedation, and use of inotropes. As ICU patients are more susceptible to develop dry eye, keratopathy, and ocular infections, they should be consulted by an ophthalmologist for early diagnosis of ocular surface disorders.

## 1. Introduction

Patients in intensive care units (ICU) are at increased risk of corneal abrasions and infectious keratitis due to impaired ocular defence mechanisms such as poor eyelid closure, inhibition of Bell's phenomenon, decreased blink reflex, reduced tear production, and increased exposure to pathogenic microorganisms [1–7].

ICU medical and nursing staff are primarily concerned with life threatening conditions; therefore the ocular signs and symptoms may be missed leading to serious ocular complications including corneal ulceration and infectious keratitis [1, 2]. Ocular complications lead to corneal opacities and even perforation which will seriously impair visual acuity and quality of life. For these reasons, meticulous eye care with regular cleaning of the eyes, installation of lubricating drops and ointments, and consultation from an ophthalmologist in case of a suspected infection [8–11] are recommended.

The aim of this retrospective study was to evaluate the prevalence of ocular surface disorders in patients who stayed in ICU more than 7 days and were consulted by an ophthalmologist.

## 2. Materials and Methods

The study was approved by the Local Ethics Committee of Meram Medical Faculty. Our ICU is an eleven-bed general unit, which accepts average of 300 patients per year. The study included patients older than 17 years, who were hospitalized in the ICU more than 7 days, with no facial or eye injuries. The medical records of 272 ICU patients who were hospitalized between February 2010 and February 2011 were reviewed.

In routine clinical practice, ICU staff examined the eyes for the presence of lagophthalmus, redness, secretion, and pupillary reflex. Ophthalmic consultation was required if patients' ICU stay exceeded 7 days or if the ICU staff suspected any eye problems. Ophthalmologists evaluated the patients for the presence of lagophthalmus, Bell phenomenon, and pupil reflex. Anterior segment examination was done with a hand-held biomicroscopic device for the presence of punctate keratopathy, corneal erosions, infectious conjunctivitis, and keratitis. Punctate keratopathy or corneal erosion was diagnosed when corneal epithelium was stained with fluorescein under cobalt blue filter. Conjunctivitis was diagnosed when purulent or mucopurulent exudate, chemosis and redness were apparent in the conjunctiva. Keratitis was diagnosed in case of an infiltration or ulcer. Schirmers I test was performed to determine the rate of tear production by inserting a Schirmers test strip into the inferior fornix, at the junction of middle and lateral third of the lower eyelid margin, for 5 minutes without topical anesthesia. After 5 minutes, the strip was carefully withdrawn and the length of wet strip was measured with a millimeter (mm) ruler. Conjunctival and/or corneal cultures were obtained by using a sterile cotton swab. The swab was directly inoculated to serum bouillon and transferred to microbiology laboratory and cultured on blood eosin methylene blue agars. These agars were incubated at 37°C for 18–24 hours. In case of positivity, an antibiotic therapy was started according to the susceptibility tests.

Routine eye care included ocular lubrication with artificial tear drops every 6 hours and ointments twice a day. Ophthalmologists altered the treatment regimen according to the status of the subjects by increasing the artificial tears in case of dry eye, corneal erosions, or lagophthalmus or adding antibiotics in case of eye infections. In case of lagophthalmus, the eyelids are closed gently by taping or tarsorhaphy.

Acute Physiology and Chronic Health Evaluation II (APACHE II) is one of several ICU scoring systems. It is applied within 24 hours of admission of a patient to ICU; a score between 0 to 71 is computed based on several measurements; higher scores correspond to a more severe disease and a higher risk of death. Simplified Acute Physiology Score II (SAPS II) is also a scoring system that measures the severity of the disease. Its score is between 0 and 163 and it predicts mortality between 0% and 100%. It describes the morbidity of a patient when comparing the outcome with other patients. These scores were routinely calculated and noted for every patient in the ICU.

The age, gender, hospitalization period, APHACE II scores, SAPS II scores, the presence of mechanical ventilation, inotropes, muscle relaxants, sedatives, eye findingsm, and trachea culture results were recorded.

## 3. Statistical Analysis

The prevalence of conjunctival hyperemia, conjunctival secretion, corneal staining, and culture positivity were compared in patients with and without mechanical ventilation, sedation and inotropes using Chi-Square test and Schirmers test results were compared with Student's *t*-test. A *P* value less than 0.05 was accepted as statistically significant. 

## 4. Results

The medical records of 40 patients who had a detailed ophthalmic examination during ICU hospitalization was included in the study. There were 26 men and 14 women with a mean age of 40.1 ± 18.15 years (range 17–74 years). The hospitalization periods ranged between 2 and 20 weeks (median 3 weeks). Mean APACHE II score was 24.05 ± 6.67 (range 12–40) and mean SAPS II score was 36.9 ± 22.17 (range 9–77). Twenty-two subjects (55%) had mechanical ventilation, 16 (40%) had sedation, 10 (25%), had inotropes and 3 (7.5%), had muscle relaxants. Blink reflex was negative in 12 subjects (30%), Bell phenomenon was absent in 30 subjects (75%), and pupillary reflex was negative in 2 subjects (5%). Inadequate lid closure was detected in 16 subjects (40%).

Out of 80 eyes, conjunctiva hyperemia was noted in 45 eyes (56.25%), mucopurulent or purulent secretion in 29 eyes (36.25%), corneal staining in 12 eyes (15%), and corneal filaments in 4 eyes (5%). Keratitis was observed in 4 patients (10%) and treated successfully with topical antibiotics. The rates of conjunctival hyperemia, secretion and corneal staining were similar between subjects with and without mechanical ventilation, with and without sedation, and with and without inotropes (*P* > 0.05) (Table 1). 

Mean Schirmers test results were 7.6 ± 5.7 mm/5 min (median 6.5 mm/5 min) in the right eyes and 7.9 ± 6.3 mm/5 min (median 7 mm/5 min) in the left eyes. In right eyes, Schirmers test results were ≤5 mm in 18 eyes (45%), 6–10 mm in 13 eyes (32.5%), and ≥11 mm in 9 eyes (22.5%). In left eyes, Schirmers test results were ≤5 mm in 17 eyes (42.5%), 6–10 mm in 12 eyes (30%), and ≥11 mm in 11 eyes (27.5%). No significant differences were found in mean Schirmers test results between subjects with and without mechanical ventilation (*P* > 0.05), with and without sedation (*P* > 0.05), and with and without inotropes (*P* > 0.05) (Table 1). Right Schirmers test results were ≤5 mm in 45% of patients with mechanical ventilation, and 44.4% of patients without (*P* = 1), left Schirmers test results were ≤5 mm in 45.5% of patients with mechanical ventilation, and 38.9% of patients without (*P* = 0.75). Right Schirmers test results were ≤5 mm in 50% of patients with sedation and 41.7% of patients without (*P* = 0.75) and left Schirmers test results were ≤5 mm in 50% of patients with sedation and 37.5% of patients without (*P* = 0.52). Right Schirmers test results were ≤5 mm in 30% of patients with inotropes and 50% of patients without (*P* = 0.46), left Schirmers test results were ≤5 mm in 40% of patients with inotropes and 43.3% of patients without (*P* = 1).

Out of a total of 40 cultures from the conjunctiva, 17 (42.5%) were positive for bacteria: 10 *Staphylococcus epidermidis*, 2 *Pseudomonas aeruginosa*, 2 *Acinetobacter baumannii*, 1 *Staphylococcus haemolyticus*, 1 *Klebsiella*, and 1 *Proteus mirabilis*. Conjunctiva culture positivity did not differ between subjects with and without mechanical ventilation, with and without sedation and with and without inotropes (*P* > 0.05, all) (Table 1). Corneal and conjunctival culture was positive for *Pseudomonas aeruginosa* and *Acinobacter baumannii *in 2 of the patients with keratitis and both of the subjects had tracheal culture positivity. Blood culture was positive in only one subject without mechanical ventilation and trachea culture was positive in 4 subjects, 2 with mechanical ventilation and 2 without.

## 5. Discussion 

Ocular surface disorders have been reported to occur in up to 60% of critically ill patients [1, 2]. Patients in ICU often have impaired ocular defence mechanisms as a result of multiorgan dysfunction, metabolic disturbances, mechanical ventilation, and unconsciousness [1, 2]. 

The eyelids are important physical barriers to trauma and infections preventing the adherence of microorganisms to the ocular surface. The sedatives and neuromuscular blockers inhibit contraction of the orbicularis oculi muscle, resulting in incomplete eyelid closure, which has been reported to occur in 20% to 75% of sedated patients in ICUs [1–4, 7]. Sedation interferes with random eye movements and inhibits Bell's phenomenon, making the eye more susceptible to nocturnal lagophthalmos [1, 2, 7, 12]. Neuromuscular blockers also abolish the blink reflex, which is one of the major ocular protective mechanisms [1, 2, 7, 10–12]. Orbital haemorrhage, lid trauma, conjunctival chemosis due to positive pressure ventilation, and facial nerve paralysis may also lead to inadequate lid closure [1, 2]. Incomplete lid closure leads to drying of the ocular surface, desiccation of the cornea epithelial cells, and corneal ulceration, with an increased risk of microbial keratitis. Lesions range from punctuate epithelial keratopathy to macroepithelial erosions, and if untreated, to corneal thinning and perforation. The reported incidence rates for superficial keratopathy and corneal abrasions are between 3–60% in the ICU patients [5–7]. In the study of Hernandez and Mannis [5], superficial keratopathy was detected in 40% of randomly selected ICU patients and 90% of those were intubated. The prevalence of corneal abnormalities was higher in patients staying in ICU for 1 week or longer. In the study of Imanaka et al. [6] ocular surface disorder was found in 28 of the 143 patients (20%) whose ICU stay exceeded 7 days, which increased with continuous sedation and neuromuscular blockade. Out of 15 patients who had sedatives or muscle relaxants administered continuously for more than 48 hours in the ICU, nine patients (60%) developed corneal erosion [6]. In the study of Mercieca et al. [7], 42% of ICU patients had some degree of keratopathy, which was detected in the majority in the first week of their stay. The presence of ocular surface disease was closely correlated with the degree of lagophthalmos, which in turn was closely related to the depth of sedation or paralysis. In this study, the rates of conjunctival hyperemia, remarkable corneal staining, and corneal filaments were 56.25%, 15%, and 5%, respectively. We could not find any significant differences in conjunctival hyperemia, secretion, and corneal staining according to mechanical ventilation, sedation, and use of inotropes.

Tears lubricate the ocular surface and wash away debris and organisms. They contain antimicrobial substances including secretory immunoglobulin A, lysozyme, lactoferrin, ceruloplasmin, and complement components. In this study, decreased tear production (less than 10 mm/5 min) was found in 70% of the subjects and in 40% of the subjects; the Schirmers test results were less than 5 mm/5 min. Paralyzing and sedating agents, atropine, antihistamines, and tricyclic antidepressants were shown to decrease tear fluid production [1, 2, 10–12]. In this study, we could not find a difference in Schirmers test results according to mechanical ventilation, sedation, and use of inotropes (*P* > 0.05). As the number of subjects treated with muscle relaxants was small (3 patients), a statistical comparison could not be done. 

Patients in ICU are more exposed to pathogenic microorganisms with significant antimicrobial resistance resulting from the widespread use of multiple antibiotics, which also increases the risk of conjunctivitis and keratitis [3, 4]. Conjunctivitis is a common complication within the ICU setting and without the necessary care; this condition can spread rapidly among the patients [1, 2, 6, 13, 14]. In the study of King et al. [13], *Pseudomonas aeruginosa* was recovered from the conjunctiva of 30 patients in a university-affiliated pediatric hospital and 70% of cases occurred in pediatric ICU (PICU) patients. In the study of Brito et al. [14], out of 1443 patients in neonatal ICU, 52 developed conjunctivitis (17.7%). Mechanical ventilation, total parenteral nutrition, orogastric tube, previous antibiotic therapy, use of CVC, and birth weight of 751–1,000 g appeared to be associated with a significantly higher risk of nosocomial infections (*P* < 0.05). Coagulase-negative Staphylococcus (36.5%) and Staphylococcus aureus (23.6%) were the most common etiologic agents isolated from cultures. In this study, the rate of mucopurulent or purulent secretion was 36.25%, respectively, and conjunctival culture positivity was 42.5%. Most of the specimens were positive for *Staphylococcus epidermidis*. The other isolated microorganisms were *Pseudomonas aeruginosa*, *Acinobacter baumannii*, *Staphylococcus haemolyticus*, *Klebsiella*, and *Proteus mirabilis*. In the study of Mela et al. [3], 54 (77%) patients were colonised by at least one bacterial species other than normal flora within seven to 42 days and their isolation was closely associated with the time period of hospitalisation. Early identification of ocular surface bacteria colonisation and the administration of topical antibiotics for prophylaxis prohibited corneal infection in these patients. In our clinical practice, antibiotic drops and ointments are used routinely in ICU patients with conjunctival hyperemia and mucopurulent/purulent secretion without waiting for the culture results.

In this study, keratitis was diagnosed in 4 patients and in 2 of them, both trachea and conjunctiva/cornea cultures were positive for *Pseudomonas aeruginosa*, and *Acinobacter baumannii*. In the literature, *Pseudomonas aeruginosa, Acinetobacter spp., Staphylococcus aureus, Staphylococcus epidermidis, Haemophilus influenzae, and Streptococcus species *were shown to cause microbial keratitis [3, 4, 15, 16]. Among these microorganisms, the most common one is *Pseudomonas aeruginosa*, which is highly virulent and causes a rapid onset devastating infection [4, 15–17]. Kirwan et al. [4] reported 3 cases of microbial keratitis in ICU. *Pseudomonas aeruginosa* was isolated in 2 cases and *Acinetobacter calcoaceticus *in one patient. In 2 of the subjects, the microorganisms were also isolated from the respiratory tract. Hilton et al. [15] reported that 10 nosocomial eye infections occurred in three ICU during an 18-month period. Nine patients were intubated, all were obtunded, and all had copious sputum production. The bacteria isolated from the patients' sputum samples and from the eyes were identical in nine patients. *Pseudomonas aeruginosa* was the cause in six of the subjects with complications (three corneal ulcers, two hypopyon, one opaque cornea, and two corneal rupture).

Eye care is a very important part of nursing care in sedated and ventilated patients in the ICU. Our standard eye care in ICU patients include ocular lubrication with artificial tear drops and ointments, and topical antibiotics when needed. Ophthalmic consultations are routinely done in ICU subjects who are hospitalized for more than one week or if ICU staff suspects any eye problems. In case of lagophthalmos, monitoring of eyelid closure needs to be carefully performed as incomplete closure which may be unrecognized under the eye patches. Incomplete eye closure might lead to drying of the ocular surface, corneal epithelium abrasion, infectious keratitis, melting of the cornea, and even perforation, which might lead to loss of vision. Closure of the eyelid is most effectively done by taping. However, tarsoraphy might be needed in some cases. Prophylactic use of antibiotic ointment might be helpful in avoiding ocular surface drying and preventing secondary infection. With our standard eye care, the rate of keratitis was only 10% in high risk patients, and all of the subjects had been treated successfully with topical antibiotics. However, there is still no standard nursing eye care in ICUs and the practice varies greatly in terms of the frequency and method of eye care.

The main limitations of this study are the retrospective design and the small sample size. In this study, the prevalence of dry eye with Schirmers test results less than 5 mm was around 40% in ICU patients and ocular signs were found at least in half of the subjects. However, we only included 40 patients out of 272 who stayed in ICU more than 7 days, consulted by an ophthalmologist for a suspected ocular problem and had a detailed ophthalmological examination, which might cause a bias giving higher rates of ocular complications. We also did not evaluate the improved outcomes or quality of life with regular ophthalmology consultations, which also necessitates a further investigation. 

## 6. Conclusion

As ICU patients are more susceptible to develop dry eye, keratopathy, and ocular infections, they should be consulted by an ophthalmologist for early diagnosis of ocular surface disorders.

## Figures and Tables

**Table 1:** The rates of conjunctival hyperemia, conjunctival secretion, corneal staining, conjunctival culture positivity, and Schirmers test values (MD ± SD) in ICU patients.

	Conjunctival hyperemia % OD/OS	Conjunctival secretion % OD/OS	Corneal staining % OD/OS	Schirmers values mm/5 min OD/OS	Culture positivity %
Mechanical ventilation					
Yes	63.6/59.1	50/45.5	18.2/18.2	6.7 ± 4.4; 7.1 ± 5.6	45.5
No	50/50	22.2/22.2	11.1/11.1	8.7 ± 6.9; 8.9 ± 7	38.9
*P* values	0.52; 0.75	0.1; 0.19	0.67; 0.67	0.27; 0.36	0.75
Sedation					
Yes	68.8/68.8	50/43.8	18.8/18.8	6.1 ± 4.3; 6 ± 4.2	31.2
No	50/45.8	29.2/29.2	12.5/12.5	8.6 ± 6.4; 9.2 ± 7.1	50
*P* values	0.33/0.2	0.21/0.50	0.67/0.67	0.18; 0.11	0.33
Inotropes					
Yes	70/70	40/40	30/30	7.1 ± 4.7; 6.8 ± 4.5	40
No	53.3/50	36.7/33.3	10/10	7.8 ± 6.0; 8.3 ± 6.8	43.3
*P* values	0.47/0.46	1/0.72	0.15/0.15	0.74; 0.52	1
